# Identification of Molecular Signatures in Neural Differentiation and Neurological Diseases Using Digital Color-Coded Molecular Barcoding

**DOI:** 10.1155/2020/8852313

**Published:** 2020-09-12

**Authors:** Debora Salerno, Alessandro Rosa

**Affiliations:** ^1^Center for Life Nano Science, Istituto Italiano di Tecnologia, Rome 00161, Italy; ^2^Department of Biology and Biotechnology Charles Darwin, Sapienza University of Rome, Rome 00185, Italy

## Abstract

Human pluripotent stem cells (PSCs), including embryonic stem cells and induced pluripotent stem cells, represent powerful tools for disease modeling and for therapeutic applications. PSCs are particularly useful for the study of development and diseases of the nervous system. However, generating *in vitro* models that recapitulate the architecture and the full variety of subtypes of cells that make the complexity of our brain remains a challenge. In order to fully exploit the potential of PSCs, advanced methods that facilitate the identification of molecular signatures in neural differentiation and neurological diseases are highly demanded. Here, we review the literature on the development and application of digital color-coded molecular barcoding as a potential tool for standardizing PSC research and applications in neuroscience. We will also describe relevant examples of the use of this technique for the characterization of the heterogeneous composition of the brain tumor glioblastoma multiforme.

## 1. Introduction

Pluripotent stem cells (PSCs) can be derived directly from the embryo at the blastocyst stage (embryonic stem cells (ESCs)) or from adult cells by reprogramming (induced PSCs, iPSCs). Regardless of the origin, these cells can be virtually converted into any cell type for basic and translational research. Reprogramming from patients allows generating iPSCs carrying disease mutations, and recent advancement in genome editing techniques has greatly facilitated the generation of mutant PSCs. In particular, the CRISPR/Cas technology is now routinely used for introducing or correcting pathogenic mutations in ESCs and iPSCs. Moreover, development of improved differentiation protocols allows efficient conversion of PSCs into disease-relevant cell types. Such remarkable advancements are mirrored by an expanding toolbox of techniques that aim capturing the transient changes in the transcriptome during differentiation. In this review, we focus on the applications of digital color-coded molecular barcoding for gene expression analysis of PSCs during neural differentiation and in neurological disease models. We also show how this technique can help improving the characterization of glioblastoma multiforme, a brain tumor showing cellular and molecular heterogeneous composition.

### 1.1. Neural Differentiation of Pluripotent Stem Cells

Human ESCs (hESCs), derived from blastocysts produced by *in vitro* fertilization for clinical purposes, have been described for the first time in 1998 [1]. It became immediately clear that their remarkable replicative capacity and plurilineage developmental potential represented a promise of unlimited supply of specific human cell types. Three years later, the first neural progenitors were obtained by spontaneous differentiation of hESCs *in vitro* [2]. Differentiation was triggered by simply culturing hESCs in suspension in the absence of feeder cells, as floating embryoid bodies (EBs). Plated EBs developed in characteristic structures that resembled the early neural tube, named neural rosettes. Cells of the neural rosette expressed characteristic neural precursor markers, such as NESTIN, Musashi-1, SOX1, and PAX6 [2–5]. Isolated neural precursors could be expanded in suspension culture as cell aggregates called neurospheres. Further differentiation generated all three central nervous system (CNS) cell types *in vitro*: neurons (mostly glutamatergic), astrocytes, and oligodendrocytes [2]. Notably, hESC-derived neural precursors engrafted into the lateral ventricles of newborn mice migrated, incorporated, and differentiated *in vivo* [2]. As a next step, several groups developed useful protocols for generating individual CNS cell types from ESCs. Human neuron subtypes generated from hESCs, including midbrain dopaminergic neurons [3, 4] and spinal motor neurons [5], became readily available. Since PSCs are pluripotent, the efficiency of conversion into a desired cell type is usually low, as many cells in culture undertake alternative differentiation pathways. A major advancement came from a novel approach leading to highly efficient neural induction. Building up from the notion that vertebrate embryonic cells differentiate by default into nerve cells in the absence of TGF*β* signaling [6], the Studer lab obtained rapid and complete neural conversion of hESCs under adherent culture conditions by simultaneous blocking the two canonical branches of the TGF*β* signaling (dual-SMAD inhibition) [7]. This method allows directed conversion of PSCs into neural precursors by blocking alternative lineages. Today, dual-SMAD inhibition represents the first neural induction step in most methods used for obtaining CNS cells from human PSCs. Upon neural induction, PSCs undergo a default anterior neural specification pattern, which can be diverted by extrinsic cues to instruct regional patterning along rostro-caudal and dorsoventral axes, producing a wide variety of neuronal subtypes [8].

### 1.2. Pluripotent Stem Cells for Neurological Disease Modeling

Similar to hESCs, human iPSCs (hiPSCs) can be virtually converted into any cell type (pluripotency) and are endowed with unlimited self-renewal capacity (stemness). iPSCs with pathogenic mutations, either obtained from patients or modified by genome editing, represent a powerful tool for advancing our knowledge on the fundamental mechanisms underlying molecular and functional human nervous tissue homeostasis and disease. HiPSCs have been used to model several diseases of the nervous system, including amyotrophic lateral sclerosis (ALS) [9–11], spinal muscular atrophy (SMA) [12], Alzheimer's disease (AD) [13], Huntington's disease (HD) [14], Parkinson's disease (PD) [15], and Fragile-X syndrome (FXS) [16]. Modeling of neurological disease requires the production of physiological *in vitro* models. A major advancement in this direction has been provided by the development of three-dimensional (3D) models of the nervous system with iPSCs. Brain organoids are 3D structures built by self-organization of differentiating hiPSCs and recapitulate, to some extent, the organization of the human brain and the variety of the cell types contained in it [17]. Brain organoids derived from hiPSCs have been successfully used to model several neurodevelopmental diseases, including microcephaly, Miller-Dieker syndrome, Lissencephaly, Timothy syndrome, and Zika virus infection [18–25]. Recent development of 3D bioprinting technologies provides new opportunities in the field of brain disease modeling with hiPSCs [26].

A major common limiting factor of current brain disease models is the quality of hiPSC-derived cells, which are often not fully representative of their physiological counterparts and include poorly differentiated cells and/or undesired lineages. It is well-documented that cells derived from hiPSCs differentiation often exhibit functional, structural, and metabolic features more similar to foetal or neonatal cells. As an example, differentiation into motor neurons generates mixed populations are often limited to the spinal subtype and represent an immature embryonic stage [27–29]. These have become major and common obstacles to hiPSCs application in modeling and treating late-onset neurological disorders. Moreover, significant variability has been observed in the quality and organization of different brain regions in individual organoids [17, 30]. Thus, improved techniques that allow better characterization of the transient transcriptional landscape of individual cells in hiPSC-derived brain models of human diseases are highly demanded.

### 1.3. Digital Color-Coded Molecular Barcoding

Among the advanced molecular biology platforms for gene expression analysis and noncoding RNAs (ncRNAs) detection, such as microarrays and high-throughput sequencing, the NanoString Technologies nCounter Analysis System (NanoString Technologies, Seattle, WA98109, USA) is one of the interesting technologies offering high levels of precision and sensitivity, achieving reproducible results and minimizing hands-on time during the experimental setting and data analysis [31]. The NanoString nCounter platform is based on a high-throughput, multiplexed, fluorescence-based digital hybridization technology, suitable for any type of nucleic acid, and therefore, it can be employed for mRNA analysis, genomic mutations, ncRNA expression, and fusion transcripts identification as well as protein levels detection [32, 33].

The detection of molecular signatures, in terms of gene expression profiling and/or ncRNAs expression, represents a potential goal to identify molecular mechanisms in neural differentiation and in neurological diseases, including tumors, developmental, and degenerative disorders. Because most neurological disease diagnosis rely on postmortem confirmation of pathologies and/or on medical imaging during their progression, it is necessary a valid approach to screen a large number of potential markers in a timely and cost-effective manner allowing an early diagnosis. The NanoString technology satisfies these requirements and moreover can be applied to critical samples, such as Formalin-Fixed Paraffin-Embedded (FFPE) and single cells derived from neural cell lines and PSCs.

The automated nCounter NanoString technology utilizes an innovative digital color-coded barcode method detecting and counting hundreds of unique transcripts in a single reaction. This technology is based on hybridization of fluorescent barcodes to specific nucleic acid sequences, in order to measure up to 800 targets for each sample, starting from low amount of material without amplification steps [34]. In particular, the system uses the reporter probes (short and gene-specific probes), characterized by different combinations of four distinct fluorophores at six contiguous positions; this approach allows to obtain a large diversity of color-based barcodes, each one specific for a gene transcript, that can be mixed together in a single tube reaction for hybridization step and individually identified in the data analysis [35]. A combination of reporter probes and capture probes (biotinylated) makes up a CodeSet that provides a handle for the attachment of molecular targets facilitating downstream digital detection [31, 33]. After hybridization, the excess unbound probes are washed away, and the molecular barcodes, covalently linked to the gene-specific probe sequences in a translucent cartridge, are quantitatively counted using an automated digital scanner (nCounter Digital Analyzer). The raw counts are first normalized for both positive and negative internal controls and for housekeeping genes and then compared within and across samples to obtain the expression of each target [33].

Gene expression analysis using a specific population of cells is an important goal to understand the intracellular molecular mechanisms underlying each cell subtype particularly in the context of neurological diseases, where a specific subset of cells is affected by the different pathologies [36]. In particular, in many neurodegenerative disorders, such as AD, some populations of neurons result vulnerable while others unaffected; for this reason, it is important to evaluate the gene expression profile at a single cell level, e.g., examining human neuronal and glial cells derived from AD iPSCs [37]. The nCounter Single Cell Gene Expression assay allows the gene expression profiling from single cells or from quantities as small as 10 pg of total RNA. In this case, due to the low amounts of mRNA from each single cell, the method requires a preamplification step using specific pairs of multiplexed target enrichment primers (MTE primers) [38].

## 2. mRNA Signatures

### 2.1. mRNA Signatures in Neural Differentiation

**Figure 1:**
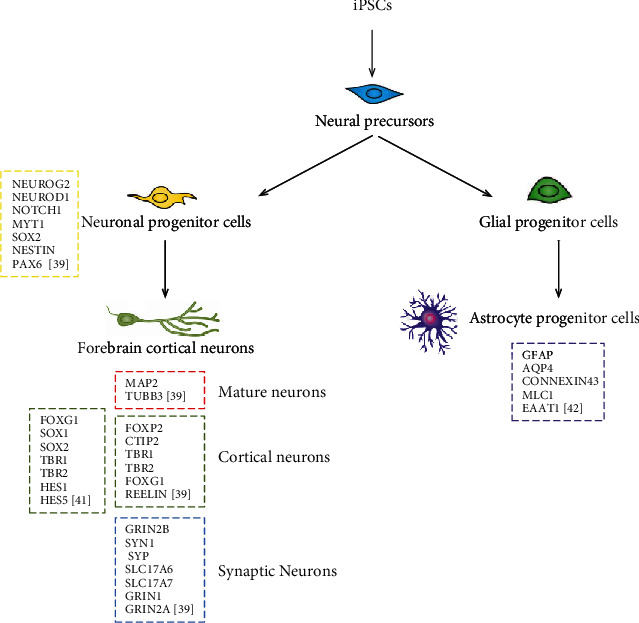
Molecular barcoding gene signatures in human PSC neural differentiation. Schematic representation of the major lineages generated from human PSCs upon neural differentiation. Gene expression signatures identified by digital color-coded barcoding are indicated. References are indicated.

Stem cell-based neuronal differentiation is frequently used to generate *in vitro* models of neuronal development and disease [39, 40]. Due to the magnitude of research dedicated to understand the gene expression of ESCs and iPSCs, it is important to identify a molecular signature in the different stages of neural differentiation for screening drugs and cell therapies for various diseases. The combination of PSCs for neural progenitor cell (NPC) generation techniques and digital color-coded barcoding for a gene expression profiling has been described (Figure 1). In particular, by using different methods for neuron generation (neuroectoderm and neurosphederm methods) from ESCs and iPSCs, sets of specific neuronal genes from the progenitors (e.g., NEUROG2, NEUROD1, NOTCH1, MYT1, SOX2), mature neurons (MAP2, TUBB3), cortical neurons (e.g., FOXP2, CTIP2, TBR1), and synaptic neurons (e.g., GRIN2B, SYN1, SYP) have been characterized [39]. Moreover, hiPSC-derived forebrain cortical neurons have been well defined by gene expression analysis, showing a robust expression of forebrain cortical transcription factors (FOXG1, SOX1, SOX2, TBR1, TBR2, HES1, HES5) with negligible expression of midbrain and hindbrain transcription factors (EN1, HB9, HOXB6, HOXB13) [41]. Furthermore, astrocyte progenitors from hiPSCs and hESCs transplanted into the ventral horn of the adult rodent spinal cord have been characterized by *in vivo* gene expression analysis; in particular, structural (such as GFAP) and functional (AQP4, CONNEXIN43, MLC1, EAAT1) astrocyte genes have been defined [42].

### 2.2. mRNA Signatures in in Neurological Disorders

**Figure 2:**
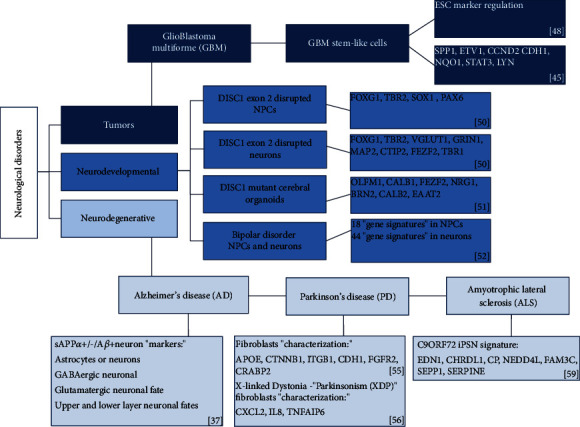
Molecular barcoding gene signatures in diseases of the nervous system. The figure shows gene expression signatures identified by molecular barcoding in several neurological diseases. References are indicated.

As mentioned, molecular barcoding represents an important approach for molecular signatures identification in neurological diseases, including tumors, disorders of development, and degenerative disorders. The scientific studies based on this technology are providing results to a more complete understanding of neurological disorders and their treatment.  Figure 2 summarizes the current scientific evidences concerning nCounter gene expression profile in neurological diseases.

Microglia, the resident immune system macrophages in the brain and cerebrospinal fluid, plays a specific neuroinflammation role in both the normal CNS functions and the neurodevelopmental and/or neurodegenerative diseases progression and resolution; its molecular barcoding signature in induced microglia-like cells represents an important characterization to understand microglia biology in order to target it in the treatment of CNS disease [43, 44]. In particular, the human-induced microglia-like cells (hiMGs) show a very similar expression pattern to foetal primary microglia, characterized by genes highly and/or uniquely expressed in human microglia (P2RY12, C1QA, GAS6, MERTK, GPR34, and PROS1). A similar trend was observed in the microglia microRNA (miRNA) signature as described below [44]. Moreover, Butovsky et al. [43] demonstrated that murine microglia signature is unique in adult microglia cells and that the ESC-derived microglia displays the same gene modulation respect to newborn and primary microglia [43].

#### 2.2.1. mRNA Signatures in Neurological Tumors

About the brain tumors, the glioblastoma multiforme (GBM) represents an important model for gene and miRNA expression evaluation because it is characterized by heterogeneous mixture of cellular and molecular subtypes [45]. Several research data have demonstrated that glioblastoma cells retain many of the features of neural progenitor cells, described as GBM stem-like cells (GSCs), and four molecular subtypes of glioblastoma are identified: proneural, neural, classical, and mesenchymal [45–47]. A GSC molecular barcoding characterization has demonstrated a transcriptional regulation of ESC markers, where NANOG, OCT4, and SALL4 genes show relatively low expression and STAT3 and SOX2 genes display high levels of expression [48]. Moreover, in a xenograft study performed by Garner et al. [45], the adherent GSCs isolated from GBM show a NanoString molecular signature characterized by downregulated (SPP1, ETV1, CCND2) and upregulated (CDH1, NQO1, STAT3, LYN) gene set [45]. Besides, a recent study displays a molecular signature of live quiescent GBM (qGBM) cells and their proliferative counterparts (pGBM) in order to identify GBM molecular subtypes. The digital color-coded barcoding transcriptome analysis reveals a mesenchymal shift as a general feature of qGBM cells: pathway scores for ECM (Extra Cellular Matrix) structure, EMT (Epithelial Mesenchymal Transition), and Cell Adhesion were increased in qGBM relative to pGBM counterparts, indicating that qGBM cells undergo a general shift towards increased mesenchymal features [49].

#### 2.2.2. mRNA Signatures in Neurodevelopmental Disorders

About neurodevelopmental disorders, the molecular barcoding gene expression analyses in stem cells have been conducted on DISC1 gene, implicated in several neurodevelopmental processes (proliferation, synaptic maturation, neurite outgrowth, and neuronal migration), and represented by multiple isoforms [50]. In particular, the effects of DISC1 disruption, limited to exon 2 and exon 8, in NPCs and neurons derived from hiPSCs have been evaluated. In both DISC1 exons disrupted NPCs, a significant decrease of FOXG1 and TBR2 expression has been observed, but only exon 2 disrupted NPCs displayed a modulation of SOX1 and PAX6 genes. The FOXG1 and TBR2 expression decreasing has been confirmed also in DISC1 disrupted neurons; in addition, the exon 2 disrupted neurons showed low levels of mature neuronal genes (VGLUT1, GRIN1, MAP2) and a decreasing expression of the cortical neuronal markers (CTIP2, FEZF2, TBR1), while exon 8 interruption did not significantly alter neuronal layer marker expression. These data suggest that DISC1 exon 2 mutation causes more dramatic deregulation of neurogenesis than DISC1 exon 8 interruption [50]. Moreover, a molecular signature characterized by upregulated (OLFM1, CALB1, FEZF2, NRG1) and downregulated (BRN2, CALB2, EAAT2) genes has been established in DISC1-mutant cerebral organoids by using a custom NanoString panel of 150 genes related to neuronal development, maturity, and cell signaling [51].

Besides, related to neurodevelopmental disorders, a PsychGene NanoString panel has been used to establish a molecular signature in hiPSCs, NPCs, and postmitotic neurons, isolated from bipolar disorder (BD) patients (two parents unaffected and two sons affected). In the NPC gene expression analysis, eighteen genes showed significant expression differences between BD patients relative to unaffected parental controls. Among these genes, NKX2-2, NKX6-1, and IRX3 are known to function in sonic hedgehog-dependent neural patterning to specify the identity of ventral progenitor-derived neurons. Comparing expression levels in BD patient postmitotic neurons to their unaffected parental controls, forty-four genes differentially expressed have been identified. In particular, an increase in expression of the general neural differentiation markers DCX and MAP 2 and a decrease in expression of cortical layers markers (CTIP2, RELN) have been observed in affected BD patients [52].

#### 2.2.3. mRNA Signatures in Neurodegenerative Disorders

Different research data highlights a gene expression signature in neurodegenerative disorders, such as AD, PD, and ALS, by combining the use of hiPSCs and molecular barcoding approach. Using the NanoString's Single Cell method, Liao et al. [37] established a molecular signature in single living iPSC-derived neurons with different secretion of insoluble extracellular amyloid *β* (A*β*) and soluble amyloid precursor protein-alpha (sAPP*α*). In particular, A*β* and sAPP*α* analytes are crucial to AD pathogenesis, and the transcriptomic characterization of three secretion profiles (sAPP*α*-/A*β*-; sAPP*α*+/A*β*-; sAPP*α*+/-/A*β*+) has identified a specific molecular signature. Individual cells secreting high levels of sAPP*α* and/or A*β* showed an expression of astrocytes or neurons markers, an elevate expression of GABAergic neuronal markers and glutamatergic neuronal fate markers, as well as upper and lower layer neuronal fates markers [37]. Moreover, a study based on the application of the NanoString's Single Cell and hiPSCs from AD patients harbouring a dominant, fully penetrant mutation in amyloid precursor protein (APP) gene (V717I) highlighted that control and AD iPSCs showed no significant differences in terms of general neuronal or cell fate specific marker expression [53]. Other studies have been conducted on another neurodegenerative disorder, PD, a sporadic, progressive disease linked to a complex genetic architecture and environmental exposures [54] and for these reasons have been attributed to a combination of genetic and nongenetic factors [55]. In order to reduce the effect of genetic variability on the study of this pathology, a characterization of hiPSC lines derived from fibroblasts of the PD affected monozygotic twin, unaffected twin, and a subject with sporadic PD, and healthy subjects have been established; markers for three-germ layer differentiation APOE and CTNNB1 (Endoderm), ITGB1 and CDH1 (Mesoderm), and FGFR2 and CRABP2 (Ectoderm) have been evaluated using the NanoString approach [55]. Moreover, the NanoString fibroblasts characterization in X-linked Dystonia-Parkinsonism (XDP), a progressive neurodegenerative disease causing the loss of medium spiny neurons within the striatum, identified a dysregulation of gene sets. The molecular signature associated to nuclear factor-kappa B (NF*κ*B), and in particular, a strong downregulation of CXCL2, IL8, and TNFAIP6 has been observed in XDP vs. control fibroblasts [56]. Lastly, ALS molecular signature has been performed by digital color-coded barcoding approach as well. The hexanucleotide GGGGCC repeat expansion in the first intron/promoter region (noncoding region) of the C9ORF72 gene is the most common genetic cause of this pathology [57, 58]. Using 50-mer NanoString probes, the levels of the three C9ORF72 RNA variants were determined in samples of patient-derived human brain tissue, ALS fibroblasts, iPSCs, and iPSC-derived neurons (iPSNs). C9ORF72 ALS patient iPSNs showed approximately a 50% reduction in expression of C9ORF72 V1 and V2 variants [59]. Moreover, sixteen aberrantly expressed target genes in C9ORF72 ALS patient tissues have been identified; in particular, seven displayed similar dysregulation patterns when compared to iPSNs: EDN1, CHRDL1, and CP were upregulated, and NEDD4L, FAM3C, SEPP1, and SERPINE2 were downregulated in C9ORF72 iPSNs versus control [59]. These seven genes represent an important molecular signature for ALS and can be potential candidates for establishing the disease biomarkers to monitor therapeutic approaches.

## 3. Noncoding RNA Signatures

### 3.1. microRNA Signatures in Neural Differentiation

Other to mRNAs, also miRNAs have demonstrated their potential as biomarkers for a wide variety of human pathologies. A deregulation of miRNA expression might be involved in neurological dysfunction or neurodegenerative processes [60]. However, while NanoString mRNA signatures by using ESCs and/or iPSCs have been well investigated in cell neural differentiation and in neurological disorders, little information for miRNA expression, and generally for all noncoding RNAs, is known.

A miRNA NanoString profiling over the time course of differentiation (4 days), obtained overexpressing a pair of transcription factors (Neurogenin-1 and Neurogenin-2) in iPSCs (iNGN cells), has been characterized. At day 4 of differentiation, compared to day 0, a signature of eighteen miRNAs downregulated and fifty-five miRNAs upregulated was defined suggesting a rapid change of the miRNA profiles in the course of iNGN differentiation. In particular, at day 0, the uninduced iNGN cells showed miRNA signatures of stem cells; the miR-302/367 cluster dominated their profile confirming its role in regulating self-renewal and preserving pluripotency. At day 4, the miR-124, important for neural differentiation, showed a consistent overexpression along with other neuronal miRNAs (miR-96 and miR-9), establishing neuronal miRNA signatures in iNGN cells [61].

### 3.2. microRNA and lncRNA Signatures in Neurological Disorders

As well described for mRNA signature, also digital color-coded barcoding technology represents an important approach for ncRNA signature identification in neurological diseases. However, scientific evidences concerning the triplet ncRNA-stem cell-NanoString are still remaining insufficiently characterized, and in particular, no ncRNA signature has been defined for neurodegenerative disorders.

As described for gene signatures, the identification of a unique microglial miRNA NanoString signature in hiMG represents another important goal to understand progression and/or resolution of neurological disease. As a mirror of gene expression data, a hiMG signature represented by nineteen downregulated and one hundred and eleven upregulated miRNAs has been established; this characterization suggests that hiMG most closely resembled foetal primary microglia than the microglia isolated from adult postmortem brain tissue, showing instead an opposite profile [44].

#### 3.2.1. microRNA and lncRNA Signatures in Neurological Tumors

Among neurological tumors, as well as for mRNA signature, the scientific evidences about ncRNA signatures are predominantly in GBM. Importantly, GSC subtype classification was demonstrated by signatures of long noncoding RNAs [47], instead miRNAs have not been shown to predict GBM classification and prognosis by global signature, while being strongly implicated in GBM [62]. In particular, a lncRNA NanoString study revealed a signature in GBM tissue and in GSCs from GBM specimens: fifteen lncRNAs resulted deregulated in GBM tissue, when compared to adjacent tissue, and twenty-seven lnRNAs showed a differentially expression in different subtype of GSCs. The lncRNA HIF1A-AS2 (hypoxia-inducible factor 1 alpha-antisense RNA 2) was significantly enriched in both GBM tissue and in mesenchymal GSCs [47]. Concerning miRNA molecular expression, a NanoString study conducted on two nonmalignant neural stem cells (NSCs) and eight GSC samples showed a signature characterized by four miRNAs downregulated in GSCs: miR-15a, miR-30c, miR-128, and miR-328 [46].

#### 3.2.2. microRNA Signatures in Neurodevelopmental Disease

Related to the ncRNAs NanoString signature in neurodevelopmental disorders, miRNA expression pattern in NSCs was determined considering Autism Spectrum Disorders (ASD) as a model. Generally, the neurodevelopmental disorders are caused by a wide variety of mutations in genes involved in protein translational control, chromatin modification, and cell division and differentiation, such as MBD5 (Methyl-CpG binding domain 5) and SATB2 (Special AT-rich binding protein 2), critical genes in ASD. In particular, an nCounter miRNA expression pattern was evaluated in NSCs in which short hairpin RNA was stably incorporated to suppress MBD5 and SATB2 and in proliferating and differentiating NSCs. A miRNA signature for MBD5 KO and SATB2 KO was established (twenty-one and thirty-one miRNAs, respectively), and interestingly, four miRNAs associated with differentiation or suppression of proliferation (miR-99, miR-9, miR-30b, and miR-92a-3p) were unregulated in MBD5 KO and in differentiating NSCs; while for SATB2 KO, all thirty-one miRNAs (in addition to miR-99, miR-9, miR-30b, and miR-92a-3p, also let-7e, miR-221-3p, and miR-93-5p) showed a significant overlap with the same trend of differentiating rather than proliferating NSCs [63].

## 4. Conclusions and Future Perspectives

The use of digital color-coded molecular barcoding in PSC-based models has the potential of greatly improving our ability to capture signatures of human development and diseases. This is particularly important for the nervous system. It is indeed crucial to improve our knowledge of the complexity and variety of the cell types that make our brain to understand the pathophysiology of neurological diseases. The molecular color-based barcoding approach offers several key advantages including precision, sensitivity, reproducibility, technical robustness, absence of an amplification step and direct measurement of target molecules, and data analysis easiness [31]. However, this novel technology requires expensive equipment (instruments and experimental kits) distributed by only one company, resulting in a closed platform and may not be cost-effective for low number of samples [64]. As described in this review, digital color-coded molecular barcoding generates robust results in PSCs, in terms of gene and ncRNA expression; however, to date, molecular information regarding ncRNAs, specifically lncRNAs, are very limited. Definition of specific molecular signatures will allow developing novel therapeutic approaches and design targeted treatments. Given the importance of the nonprotein coding genome in the human nervous system, we expect for the future an increase on the use of molecular barcoding for the characterization of long and short noncoding RNAs expressed in brain cells under physiological or pathological conditions.

Given that many neurological diseases, in particular neurodegeneration disorders, manifest pathologically as proteopathies [65], more emphasis should be placed on direct detection of protein levels in disease models. It would be particularly informative to investigate on genes-proteins association from the same sample, in order to exhaustively study neurological disease pathogenesis, highlighting the importance of combination of genetic and biochemical analyses. For this reason, it would be interesting to take advantage the 3D Biology™ system proposed by NanoString, which allows evaluating RNA, DNA, and proteins in a single assay, allowing a 360 degree view on the profiling of a neurological disease. Another interesting future perspective could be the spatial and simultaneous resolution of RNAs and proteins on a single platform and digital counting of both analytes from a single sample in order to establish a tissue “geography” (Digital Spatial Profiling).
